# New-York Correspondence

**Published:** 1873-01

**Authors:** W. A. Mitchell

**Affiliations:** Glennville, Alabama.; New York City


### NEW YORK CORRESPONDENCE.

New York City, January 25, 1873.

*Dr. W. F. Westmoreland, Atlanta, Georgia:*

My Dear Doctor,—In accordance with my promise, I send you a few clinical items, which I trust may be of interest to the readers of your journal. You having agreed to bear all the blame, should these items be pronounced unsatisfactory, I shall make no apology for lack of time to prepare them better.

In the first place, you doubtless remember the little O'Farral boy, twelve years of age, whose left hip-joint Dr. L. A. Sayre excised while you were here. The points I have been able to collect of his history are these:

Enjoying ordinary health up to the age of eight, he then began to develop those symptoms indicative of hip-joint trouble. An apparatus was adjusted, with manifest improvement; but an unfortunate fall, which did great violence to the joint, set up all the inflammatory processes anew, finally reducing him to the condition in which we saw him: emaciated, pale, exhausted; pulse 120, temperature 103; countenance painfully wearied and pinched; left leg drawn up and supported by its fellow in the usual manner, with a sinus leading from the cotyloid cavity down midway the external aspect of the thigh, and there discharging daily more than ounce of fœtid, greenish looking pus. Dr. Sayre, introducing his knife into the external opening of this sinus, laid it open to the joint, and continued the incision to a point just below the anterior superior spinous process of the ileum, thus freely exposing the joint. A good deal of the head of the femur was destroyed; but he sawed off what was left, together with the neck, having first peeled off the periosteum. This last process, Dr. Sayre remarked, was easily accomplished upon dead bone, but very difficult on healthy bone.

Having gouged out the acetabulum, and thoroughly cleansed the wound, he closed the original incision with interrupted silver sutures. Then, entering his knife in the line of this incision immediately over the joint, he made another cut downward and backward for two inches, to allow free drainage; and to facilitate this, he stuffed oakum into the joint through this wound; lint, he said, should never be used in this way, as he has seen bad consequences ensue from retention of small particles of it in the wound. The Doctor then put the child in his beautiful and ingenious apparatus which he invented for such cases, and by the use of which the little patient may be carried from room to room, up or down stairs, and out into the pure air and sunshine, while the limbs and pelvis are kept perfectly immovable. Any one can readily see how much greater are his chances of life, than if compelled to lie in bed during the time necessary for his recovery. His pulse and temperature the very day after the operation, was below a hundred, and he has continued to improve daily, so you would scarcely know him now.

Day before yesterday he performed the same operation on a little patient somewhat younger than the one just mentioned. He was doing well this morning, and everything indicates a favorable result. Some inquiry being made, after the operation, as to the future usefulness of such limbs, the Doctor said most of them were useful, and some wonderfully perfect. He drove us around to see a young gentleman upon whom he excised the head and neck of the femur eight years ago. He was not in; supposed to be out at Central Park *skating*, in which sport he was so efficient as to win a pair of silver skates in a skating match, in which were engaged some forty contestants.

Dr. Sayre is remarkably successful in his cases of anchylosis, which he breaks thoroughly and completely at one sitting; not a little to-day and the remainder to-morrow, but all at once, and then bandages the limb well from the extremity up to the body; and when he gets above the affected joint, say the knee, for example, he takes a piece of sponge somewhat larger than an egg, and placing it over the femoral artery in its most superficial portion, carries his bandage over it, pressing it with sufficient firmness to lessen the capacity of this artery and diminish the amount of blood usually going to the part, though, owing to the elasticity of the sponge, enough is allowed to pass to keep up the nutrition of the part. The joint is to be kept immovable, elevated, and extension practiced until the danger of inflammatory action is passed. Should this occur, however, he considers ice-cold applications the best treatment. I would like to give you an account of the Doctor's different operations and appliances for each form of club foot, but as it would require a small book to do this, I shall not attempt it in a short letter.

Dr. J. Marion Sims has shown me various interesting operations. In one case he removed the coccyx of a young lady who had suffered for years from coccydynia. The operation is simple enough; but Dr. Sims remarked that if the wound was not carefully treated, an abscess would ensue, which from its site and nature was somewhat difficult to cure.

I also saw him excise, as he terms it, nearly the entire uterus, for malignant disease, with an excellent result. During this operation an incident occurred which may be of interest. The patient, who was under an anæsthetic, suddenly ceased to breathe nor could pulsation be felt. The Doctor asked hurriedly whether ether or chloroform had been administered, and being answered chloroform, he seized the woman around the waist, and dragging her off of the table, allowed her head and shoulders to hang down nearly to the floor, almost standing her on her head, as it were. She very soon gasped, sighed, and directly began to respire regularly and freely.

Now the Doctor contends, that as ether is a stimulant to and chloroform a depressor of the heart's action, the latter is much more dangerous than the former; and that when death occurs from its use, it is from lack of blood in the brain, as in fatal syncope. Acting from these premises, he had several times experienced the same happy result, by causing the patient to assume the above mentioned position.

Now while Drs. Hamilton and Wood coincide with Doctor Sims, as to the superiority of ether over chloroform, Drs. Sayre and T. Gaillard Thomas entertain entirely different views, and act accordingly.

While such eminent gentlemen entertain such dissimilar views on points which should have been definitely settled long since, how can one expect the lesser lights to be all homogeneous and serene?

Among the many instructive operations of Dr. Emmet, I will mention one which to a gentleman of less experience might have been embarrassing. The patient was a woman, upon whom Doctor Emmet operated successfully, over a year ago, for an extensive vesico-vaginal fistula. She now came to him with stone in the bladder and chronic cystitis.

He proposed performing lithotomy, and afterwards cure the cystitis by injections, etc., the bladder being kept thoroughly washed out through this vesico-vaginal incision; this being, he said, the only true method of curing cystitis that he knew of.

In making the incision, owing to the changed anatomy of the parts from the operation performed a year previous, the circular artery was divided, which accident was of course attended by considerable hemorrhage. This being controlled, the forceps was introduced and the stone seized, but it crumbled under pressure, so that only about one-half was brought out. The instrument was reintroduced to take out the other half, but lo, it had disappeared! the bladder being perfectly empty, save a small quantity of soft sandy deposit. A thorough exploration of the bladder was then made with the sound, when it was ascertained that the other portion of the stone had escaped into one of the ureters, which were greatly enlarged and, from the same abnormal anatomy mentioned above, entered the bladder in just the angle to invite the escape of the stone into them. It was let alone, with the hope that it would reënter the bladder in the course of twenty-four hours, when it will be seized and taken charge of.

The Doctor was a little more fortunate in an operation of the same kind performed on another patient several days after the one just spoken of. Here he obtained the entire stone, which was very hard, and almost as large as a small hen egg, and had inclosed within it a common brass pin, with about the sixteenth of an inch of the pin's point exposed. The woman wonders how the pin ever got into her bladder, and so do we.

I have seen three ovariotomies: two by Dr. Peaslee, and one by Professor Thomas. In Dr. Peaslee's first case, the tumor was very large, multilocular, and adherent nearly everywhere, which adhesions it was difficult to break up. The cyst, having been partly emptied, was drawn out, and a double ligature passed through the pedicle, which, after being securely tied, was divided close to the ligature, and allowed to drop back in the pelvis. The abdominal cavity was then thoroughly sponged out, and the wound closed with interrupted silver sutures, except at the lower edge, where a tent was left in for drainage. The woman was very weak to begin with, and was two hours on the operating table. She died thirty-six hours afterward—never reacting from the shock, although stimulants, etc., were freely given.

The second case, which was operated on a few days after, was much more favorable in every respect. She is now fast recovering. Dr. Peaslee very confidently predicted the result in both cases.

There were two points of interest in Dr. Thomas' case which I may mention: first, the new anæsthetic, bichloride of mithylene, was used, and acted very satisfactorily indeed. Second, the woman had peritonitis, which fact Dr. Thomas was perfectly aware of, and in speaking of it just before operating, said he considered the circumstance rather favorable to the woman; for while it had a tendency to prevent traumatic peritonitis, it might itself be cured by the new character of inflammation set up. "Similia similibus curanter." We will see in a few days what will become of this patient.

A sort of epidemic of a most interesting nature is now present in the city, as illustrating the identity of the poison which causes puerperal fever in the parturient woman, and pelvic cellulitis after the slightest operation on the unimpregnated female. Several cases have died in the last ten days at Bellevue Hospital of puerperal fever, and nearly every slight operation performed since that time on the unimpregnated female has been followed by more or less trouble.

I saw Dr. Thomas take off an hypertrophied clitoris as large as a goose egg, about eight days ago. He did it very nicely, with the galvano-cautery, not losing over two drachms of blood, when the hemorrhage in such tumors, where the knife is used, is usually fearful. I was informed by the Doctor yesterday that this woman was dead of pelvic cellulitis.

Dr. Thomas has two other cases of ovariotomy to perform, Dr. Emmet one case; but all operations of that nature will be postponed until this atmospheric poison, whatever it may be, has passed away.

Dr. Sims intended performing a very unique operation this week on a patient who has suffered for years from prolapsus and retroversion, in spite of all the means and appliances he could think of. He intends, as soon as possible, passing a long curved canula, containing a long needle with an eye in its end, into the uterine cavity up to the anterior portion of the fundus. Then with the canula he will push the fundus upward and forward against the abdominal walls just below the umbilicus. Then pushing the needle through the uterus and abdominal parietes at this point, he will thread it with a long silver wire, and drawing it back into the uterus, bring it down through the vagina, to the labia, where he will tie on to the wire a large silver button. Then pulling on the end lying external to the abdominal puncture, he will draw this button up into the uterus, directly against the fundus; then, continuing traction, he will of course draw the uterus up until the peritoneal layer over its fundus is directly against the abdominal wall at the point indicated—viz., just below the umbilicus, where the wire passes out. Then firmly securing the wire on the outside, he will keep those peritoneal surfaces in opposition until circumscribed inflammation occurs, plastic material is thrown out, the surfaces firmly bound together, and the patient cured—nothing remaining to be done but to cut the wire outside, and remove the button from the uterus. There is no reason why it should not succeed, and I hope I may be able to give you the result in my next letter, as also an account of some of the new and instructive operations I have witnessed by Professors Wood, Hamilton, Markoe and Van Buren.

To all of the gentlemen named in this letter I am indebted for their marked kindness and courtesy. I don't know that I can end this letter better than by giving you the history of a case which Dr. T. G. Thomas related to me a few days ago:

He was hastily called, about ten days since, to see an intelligent young married woman, whom he found in a state of great excitement, and who told him the following story: That, having failed to menstruate at her regular period, some two weeks past, and thinking herself pregnant, she determined to produce abortion. Having heard that this could be accomplished by passing a wire up into her womb, she prevailed on her husband to procure her for this purpose a wire, which she said was the size of a knitting needle, and about eight inches long. This, on the night before, she passed up into the vagina, then into the womb until the external end of the wire reached the vulva; then, although this maneuvre had caused her some pain and hemorrhage, she concluded to pass it a little farther "to be certain." So, putting her finger directly against the lower extremity of the wire, she pushed it up, up, until it suddenly slipped off her finger, and could nowhere be found. She immediately attempted to rise, but experienced such dreadful pain at two points—one just below the umbilicus, the other about the middle of the lower lobe of the right lung—that she was compelled to lie down again, and spent the night in great pain and trepidation. Such was her story.

The Doctor fearing all this might be a ruse to induce him to pass some instrument into the uterus to hunt for this wire and thus bring about the result they desired, concluded to act cautiously. Passing the finger up the vagina he found the uterus in situ, and as far as he could judge, neither pregnant nor injured. But upon sweeping his finger around the cervix, he discovered a rent through the vaginal wall just to the left and very near to the uterus. Though this rent or puncture readily admitted the point of his index finger, no wire could be felt. Taking a steel sound, he passed it without dificulty up through this opening into the peritoneal cavity, thinking if he could locate this wire, he could introduce a pair of forceps and draw it out. He carefully swept this sound in every direction, and although he failed to touch the wire, he set up a fearful hemorrhage, which well-nigh exhausted the woman before it could be controlled. The Doctor then being in doubt as to what course to pursue, a consultation was called, and it was decided to perform gastrotomy next morning and hunt for the wire.

Next morning he called with his friends and instruments to perform said operation, but found the woman doing so much better, pain gone, nervousness subsided and no febrile reaction whatever, that they concluded the symptoms did not warrant so grave an operation as gastrotomy; and besides, her husband, while admitting that he procured the wire for her, now gave it as his opinion that she had lost it, and had never passed it into her vagina at all. This was the supposition also of her mother and others, though the patient adhered positively to her original statement. An anodyne was administered and the patient left for the day, the strictest quietude being enjoined. Dr. Thomas was now called from the city two days; upon his return he found the patient suffering from a terrible attack of pneumonia of the entire right lung, its starting point being the *lower border of the lower lobe.* The Doctor now felt sure that the wire must have been pushed up until its point entered the lung and set up this pneumonia, but the woman's intense febrile condition forbade any operation. She grew rapidly worse, gangrene of the lung taking place, and in death's dark shadow we recognize the reward of sin. At the post-mortem, the whole peritoneal cavity looked as if several ounces of ink had been poured into it, though there was no evidence of peritonitis. Passing his hand under the intestines, Dr. Thomas discovered a wire, one end of which rested just behind the uterus, the other end had passed under the liver, pierced the diaphragm and was resting, as was supposed, in lung tissue. I was shown the wire, which was the size of a large knitting needle, and measured exactly *seventeen inches long.*

Yours, truly, W. A. Mitchell, M.D.,
*Of Glennville, Alabama.*

